# Interferon-Induced Protein 44 Interacts with Cellular FK506-Binding Protein 5, Negatively Regulates Host Antiviral Responses, and Supports Virus Replication

**DOI:** 10.1128/mBio.01839-19

**Published:** 2019-08-27

**Authors:** Marta L. DeDiego, Aitor Nogales, Luis Martinez-Sobrido, David J. Topham

**Affiliations:** a David H. Smith Center for Vaccine Biology and Immunology, University of Rochester, Rochester, New York, USA; b Department of Microbiology and Immunology, University of Rochester, Rochester, New York, USA; c Department of Molecular and Cell Biology, Centro Nacional de Biotecnología (CNB-CSIC), Universidad Autónoma de Madrid, Madrid, Spain; d Center for Animal Health Research (INIA-CISA), Madrid, Spain; Columbia University Medical College

**Keywords:** FKBP5, IFI44, IFN responses, antiviral responses, innate immunity, signaling transduction, virus-host interactions

## Abstract

Using multiple viral systems, and performing silencing approaches, overexpression approaches, and experiments in knockout cells, we report, for the first time, that interferon (IFN)-induced protein 44 (IFI44) positively affects virus production and negatively modulates innate immune responses induced after viral infections. Moreover, IFI44 is able to rescue poly(I·C)- and IFN-mediated inhibition of virus growth. Furthermore, we report a novel interaction of IFI44 with the cellular factor FK506-binding protein 5 (FKBP5), which binds to cellular kinases such as the inhibitor of nuclear factor kappa B (IκB) kinases (IKKα, IKKβ, and IKKε). Importantly, in the presence of FKBP5, IFI44 decreases the ability of IKKβ to phosphorylate IκBα and the ability of IKKε to phosphorylate interferon regulatory factor 3 (IRF-3), providing a novel mechanism for the function of IFI44 in negatively modulating IFN responses. Remarkably, these new IFI44 functions may have implications for diseases associated with excessive immune signaling and for controlling virus infections mediated by IFN responses.

## Abstract (precis)

Innate immune responses mediated by IFN and inflammatory cytokines are critical for controlling virus replication. Nevertheless, exacerbated innate immune responses could be detrimental for the host and feedback mechanisms are needed to maintain the cellular homeostasis. In this work, we describe a completely novel function for IFI44 in negatively modulating the innate immune responses induced after viral infections. We show that decreasing IFI44 expression by using small interfering RNAs (siRNAs) or by generating knockout (KO) cells impairs virus production and increases the levels of IFN responses. Moreover, we report a novel interaction of IFI44 with the cellular protein FKBP5, which in turn interacts with kinases essential for type I and III IFN induction and signaling, such as the inhibitor of nuclear factor kappa B (IκB) kinases IKKα, IKKβ, and IKKε. Our data indicate that binding of IFI44 to FKBP5 decreased the phosphorylation of IRF-3 and IκBα mediated by IKKε and IKKβ, respectively, providing a likely explanation for the function of IFI44 in negatively modulating IFN responses. These results provide new insights into the induction of innate immune responses and suggest that IFI44 is a new potential antiviral target for reducing virus replication.

## INTRODUCTION

The innate immune system restricts virus replication. To this end, pathogen-associated molecular patterns (PAMPs), which include molecules such as glycoproteins and proteoglycans and nucleic acid motifs encoded by viruses, are recognized by pattern recognition receptors (PRRs) (1). Different PRRs recognize different viruses. For example, influenza A virus (IAV), which is a member of the *Orthomyxoviridae* family and which contains an eight-segmented, negative-sense single-stranded RNA (ssRNA) segmented genome, is mainly recognized by the membrane-associated PRRs Toll-like receptor 3 (TLR-3) and TLR-7 and cellular PRR retinoic acid-inducible gene I (RIG-I) (1). Lymphocytic choriomeningitis virus (LCMV), the prototype member of the *Arenaviridae* family, contains a negative-sense ssRNA bisegmented genome and is mainly recognized by TLR-2 and RIG-I (1). Sendai virus (SeV), a *Paramyxoviridae* member containing a negative-sense ssRNA, is sensed by TLR-7, RIG-I, and melanoma differentiation-associated (MDA) protein 5 (MDA5) (1). Lastly, vesicular stomatitis virus (VSV), a *Rhabdoviridae* member, whose genome is composed of a negative-sense ssRNA, is recognized by TLR-7 and RIG-I (1).

The recognition of viral pathogens or their products by PRRs initiates converging signaling pathways leading to the production of type I interferon (IFN-I) (alpha IFN [IFN-α] and IFN-β), type III IFN (gamma IFN [IFN-λ]), and proinflammatory cytokines (1). Production of type I and III IFNs is crucial for the induction of antiviral responses restricting virus replication, since type I and III IFNs trigger the transcription of IFN-stimulated genes (ISGs) (2), many of them having antiviral activity (3). However, excessive production of IFN and/or other cytokines can be deleterious to the host. Therefore, in contrast to the classical role of ISGs in antiviral activity, several ISGs play regulatory functions to control excessive antiviral responses (4, 5).

To induce the transcription of type I and III IFNs and proinflammatory cytokines, transcription factors such as (i) IFN-regulatory factor-3 (IRF-3) and IRF-7 and (ii) nuclear factor kappa-light-chain-enhancer of activated B cells (NF-κB) are activated (6). IRF-3 and IRF-7 activation involves the phosphorylation of these proteins by the kinases TANK-binding kinase-1 (TBK-1) and IKKε (7). This posttranslational IRF-3 and IRF-7 phosphorylation allows IRF dimerization and nuclear translocation and transcription of IFN and proinflammatory genes (8). (ii) In unstimulated cells, NF-κB dimers are sequestered in the cytoplasm by a family of inhibitors, named IκBs, which become phosphorylated. The multisubunit IκB kinase (IKK) responsible for IκB phosphorylation contains two catalytic subunits, IKKα and IKKβ, both being able to phosphorylate IκB, and IKKγ, which is a regulatory subunit (9). Phosphorylation of IκB inhibitors leads to their degradation by the proteosome, allowing NF-κB to migrate to the nucleus and activate IFN and proinflammatory cytokine expression (10). FK506-binding protein 5 (FKBP5) is an immunophilin that interacts with IKKα, IKKβ, and IKKγ and facilitates IKK complex assembly, leading to increased IKKα and IKKβ kinase activity, NF-κB activation, and IFN production (11). In addition, it was proposed previously that FKBP5 interacts with IKKε (12).

Type I and III IFNs signal through distinct receptors, but the pathways converge in the phosphorylation of the signal transducer and activator of transcription 1 (STAT1) and STAT2 factors (6), being the Janus protein tyrosine kinase 1 (JAK1) and tyrosine kinase 2 (TYK2) responsible for phosphorylation and activation of STAT1 and STAT2 (13). In addition, STAT1 is phosphorylated by IKKε during IFN signaling, with this step being critical for the IFN-inducible antiviral response (14, 15). Phosphorylated forms of STAT1 and STAT2 then associate with IRF-9 to form a heterotrimeric ISG factor 3 (ISGF3) complex (6), which translocates to the nucleus, where it binds to DNA sequences of IFN-stimulated response elements (ISREs) present in the promoter of ISGs to upregulate their transcription (2, 6).

IFI44 is a cytoplasmic protein that induces an antiproliferative state in cells and contains a green fluorescent protein (GTP)-binding domain, although it has no homology to GTPases or G proteins (16). The protein was initially found as a hepatitis C virus (HCV)-associated microtubular aggregate protein isolated from the hepatocytes of HCV-infected chimpanzees (17). IFI44 is an ISG (18, 19) and is induced after infection with different viruses such as rhinovirus (20) and papillomavirus (21). However, the biological functions and mechanism of action for IFI44 remained unknown.

In this work, we identified IFI44 upregulation in influenza virus-infected cells. We then performed experiments knocking down, knocking out, and overexpressing IFI44 to determine its effect on innate immune responses and on the replication of different ssRNA viruses such as IAV, LCMV, and VSV. Furthermore, using mass spectrometry and coimmunoprecipitation (Co-IP), we demonstrated an interaction of IFI44 with the cellular factor FKBP5. We show that in the presence of FKBP5, IFI44 overexpression leads to a decrease in the ability of IKKε and IKKβ to phosphorylate IRF-3 and IκBα, respectively, likely leading to decreased IFN responses mediated by IRF-3 and NF-κB. Notably, this is the first time that IFI44 has been reported to negatively modulate antiviral responses induced by multiple viral systems. Importantly, IFI44 could be used as a potential target to regulate innate immune responses after viral infection to control a negative exacerbated immune reaction that could have deleterious consequences for the host or to control the cytokine storm induced during viral infection that has been shown to be responsible, at least in part, for the pathogenesis of some viruses, such as IAVs (22, 23) or coronaviruses (24).

## RESULTS

### IFN-α treatment and IAV infection induces the expression of IFI44.

**FIG 1:**
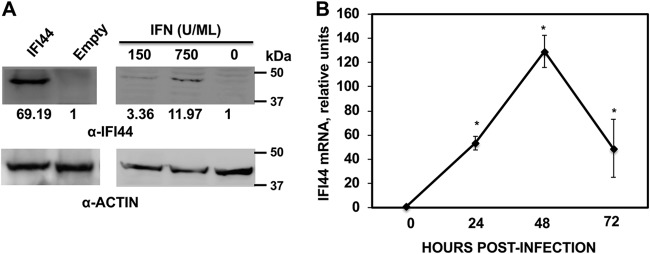
IFI44 is induced by IFN treatment and IAV infection. (A) Human 293T cells were transfected with the pCAGGS plasmid expressing IFI44-HA protein (left) or treated with 150 or 750 U/ml of universal type I IFN for 12 h (right). Western blotting was performed using anti-IFI44 (top) and actin (bottom) antibodies. Western blots were quantified by densitometry using ImageJ software (v1.46), and the amounts of IFI44 were normalized to the amounts of actin (numbers below the IFI44 blot). N.D., not detected. Molecular weight markers are indicated (in kilodaltons) on the right. Three different experiments were performed, with similar results. (B) Human A549 cells were infected with influenza PR8 virus at an MOI of 0.1. Total RNAs were collected at 24, 48, and 72 hpi, and the level of expression of IFI44 was evaluated by RT-qPCR and compared to the level seen with noninfected cells (0 hpi). Error bars represent standard deviations (SD) of results of measurements performed in triplicate wells. *, *P* < 0.05 (for comparisons between results measured for infected cells at 24, 48, and 72 hpi and those measured for noninfected cells [0 hpi] using Student’s *t* test).

In a previous transcriptomic analysis, we found that IFI44 was induced in IAV-infected cells (25). IFI44 has been previously described as an ISG (16, 18); however, its functions are mostly unknown. To further confirm that IFI44 is an ISG, human 293T cells were treated with two different concentrations (150 and 750 U/ml) of universal IFN-α for 12 h, and the levels of IFI44 upregulation were assessed by Western blotting using an antibody (Ab) specific for IFI44 (Fig. 1A). To show that this antibody is specific for IFI44, 293T cells were transfected with a pCAGGS plasmid expressing the IFI44 gene fused to a hemagglutinin (HA) epitope tag or with empty pCAGGS plasmid (Fig. 1A). The IFI44-specific antibody recognized the IFI44 protein expressed from plasmid-transfected cells as well as the endogenous IFI44 isoform in cells treated with both concentrations of IFN-α, with the IFI44 protein expression levels being higher in the cells treated with the largest amount of IFN-α (Fig. 1A). These data confirm that IFI44 expression is induced in IFN-α-treated cells in a dose-dependent manner. Next, we evaluated whether IFI44 is induced after IAV infection (Fig. 1B). To that end, human A549 cells were infected (multiplicity of infection [MOI] of 0.1) with influenza PR8 virus or were subjected to mock infection. Total RNA from PR8-infected cells was collected at 24, 48, and 72 hpi and from mock-infected cells at 0 hpi, and mRNA expression levels of IFI44 were evaluated by reverse transcription-quantitative PCR (RT-qPCR) (Fig. 1B). Increased levels of IFI44 mRNA were detected after IAV infection, reaching a maximum (up to 120-fold higher than the levels seen with mock-infected cells) at 48 hpi. These data indicated that IFN-α treatment and IAV PR8 infection induce the expression of IFI44.

### IFI44 silencing decreases virus production.

**FIG 2:**
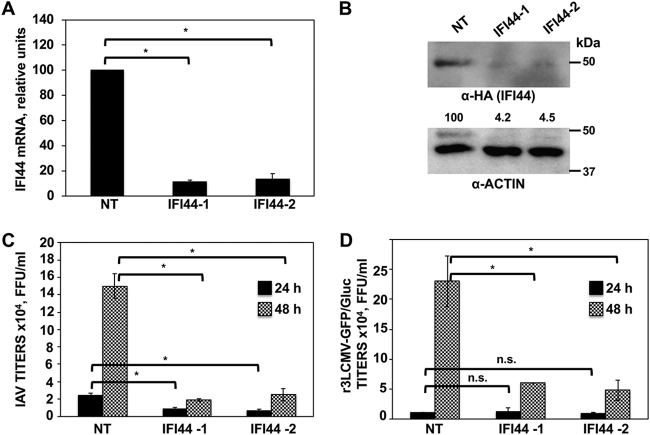
IFI44 silencing negatively affects IAV and LCMV replication. (A) Human A549 cells were transfected with NT or IFI44 siRNAs. At 36 hpt, total RNAs were purified and mRNA levels for IFI44 were analyzed by RT-qPCR. (B) At 36 h after siRNA transfection, cells were transfected for 48 h with the plasmid expressing IFI44 fused to an HA tag. A Western blot analysis using anti-HA antibodies (to detect IFI44; top) and anti-actin antibodies (bottom) was performed. Western blots were quantified by densitometry using ImageJ software. IFI44 protein expression levels in cells silenced with the NT siRNA were assigned a value of 100% for comparisons with the levels of expression in IFI44-silenced cells (numbers below the HA blot). IFI44 expression was normalized to actin expression. Molecular weight markers are indicated (in kilodaltons) on the right. Three different experiments were performed, with similar results. (C and D) At 36 hpt, cells were infected with influenza PR8 virus (C) or r3LCMV-GFP/Gluc (D). Tissue culture supernatants were collected at 24 and 48 hpi and titrated by immunofocus assay (PR8) or fluorescence expression analysis (r3LCMV-GFP/Gluc). Bars represent SDs determined using triplicate wells. Three different experiments were performed, with similar results. *, *P* < 0.05 (for comparisons between NT- and IFI44-silenced cells at 24 and 48 hpi using Student´s *t* test). n.s., differences not significant (*P* > 0.05).

To study whether IFI44 expression affects IAV production, silencing (loss-of-function) experiments were performed. Using two different small interfering RNAs (siRNAs), IFI44 mRNA was silenced in A549 cells by more than 85% as determined by RT-qPCR (Fig. 2A). To confirm that the IFI44-specific siRNAs knocked down IFI44 expression at the protein level, A549 cells transfected with the plasmid expressing IFI44-HA were silenced with the two different IFI44 siRNAs or with the nontargeted (NT) siRNA (Fig. 2B). Western blotting using an anti-HA-specific antibody showed a reduction of about 25-fold in the overall levels of IFI44 in IFI44-silenced cells using the two different specific IFI44 siRNAs (Fig. 2B). These data indicated that the IFI44 siRNAs efficiently knocked down the expression of IFI44 at the mRNA (Fig. 2A) and protein (Fig. 2B) levels.

To analyze whether IFI44 silencing had an effect on IAV production, A549 cells were silenced with the two IFI44-specific siRNAs or the NT siRNA and were then infected (MOI of 3) with PR8. Interestingly, reproducible and significant 3-fold and 7-fold reductions in PR8 viral titers were observed in the cells silenced with two different siRNAs specific for IFI44 compared to the NT siRNA-silenced cells at 24 and 48 hpi, respectively (Fig. 2C). These data suggest that IFI44 negatively affects IAV production.

To analyze whether the silencing of IFI44 negatively affects the production of other ssRNA viruses, human A549 cells were silenced with the two IFI44-specific siRNAs or with the NT siRNA control and were then infected with r3LCMV-GFP/Gluc (recombinant 3LCMV-GFP/Gluc) (MOI of 3). Notably, a reproducible and significant 5-fold reduction in r3LCMV-GFP/Gluc titers was observed in the IFI44-silenced cells compared to the NT-silenced control cells at 48 hpi (Fig. 2D). These results suggest that the negative effect of IFI44 silencing on virus replication is not limited to IAV and that it applies to other viruses, such as Old World arenaviruses.

### Effect of IFI44 silencing and overexpression on antiviral responses.

**FIG 3:**
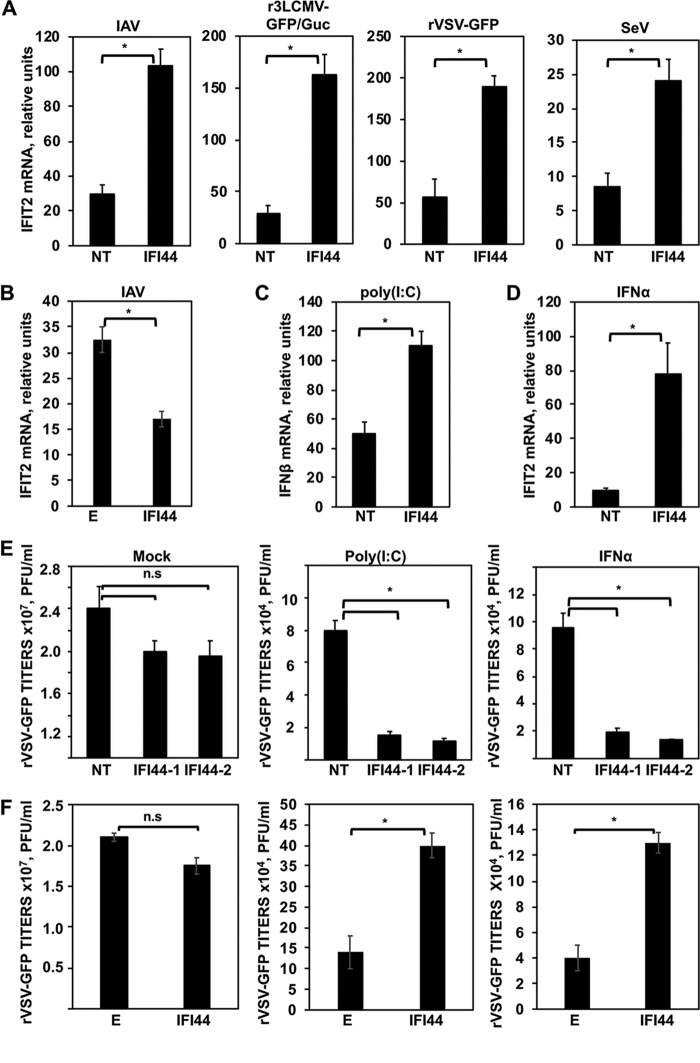
IFI44 negatively regulates host IFN responses. (A) Human A549 cells were transfected with a NT siRNA or with siRNA-1 specific for IFI44 and were then infected with IAV (MOI of 3), r3LCMV-GFP/Gluc (MOI of 3), rVSV-GFP (MOI of 0.1), or SeV (MOI of 3). (B) Human 293T cells were transiently transfected with the pCAGGS plasmids expressing IFI44 or with the empty plasmid as a control. At 36 hpt, cells were infected with IAV (MOI of 3). (C and D) IFI44 expression was knocked down in human A549 cells. Cells were transfected with poly(I·C) for 16 h (C) or were treated with IFN-α (D). (A to D) Total cellular RNA was purified, and the levels of IFN-β or IFIT2 mRNAs were evaluated by RT-qPCR. Bars represent SDs determined using duplicate wells. Three different experiments were performed, with similar results. *, *P* < 0.05 (using Student’s *t* test). mRNA levels are expressed as fold change (increases) in comparison to mock-infected or mock-treated cells, used as controls. (E) Human A549 cells were transfected with two different IFI44 siRNAs. (F) Human 293T cells were transfected with the pCAGGS plasmid expressing IFI44. (E and F) At 36 hpt, cells were transfected with poly(I·C) or treated with IFN-α to induce an antiviral state and were then infected with rVSV-GFP (MOI of 0.1). Virus production was analyzed at 24 hpi. Bars represent SDs determined using duplicate wells. Three different experiments were performed, with similar results. *, *P* < 0.05 (using Student’s *t* test). n.s., differences not significant (*P* > 0.05).

Many ISGs display antiviral responses to combat viral infection (3). However, some of them are known to regulate the antiviral response through a negative-feedback mechanism (4, 26). Taking into account that IAV and LCMV are sensitive to IFN responses (3, 27–29) and that IFI44 silencing negatively modulates the production of these viruses (Fig. 2C and D), we hypothesized that IFI44 was negatively modulating IFN responses. To evaluate this hypothesis, A549 cells were silenced with the NT siRNA control or with an siRNA specific for IFI44 and were then infected with IAV, r3LCMV-GFP/Gluc, recombinant VSV-GFP (rVSV-GFP), or SeV to induce host antiviral responses. As expected, after viral infections, high levels of IFI44 (see Fig. S1A in the supplemental material) and of IFN-induced protein with tetratricopeptide repeats 2 (IFIT2) (an ISG) (Fig. 3A) were induced. Interestingly, the levels of IFIT2 induction measured after the virus infections were 2.5-fold to 6-fold higher in the IFI44-silenced cells than in the NT-silenced control cells (Fig. 3A). Importantly, at least in the IAV-infected and r3LCMV-GFP/Gluc-infected cells, the higher level of IFIT2 induction in the IFI44-silenced cells was not due to higher virus titers, as it was found that the virus titers were lower in the IFI44-siRNA-transfected cells (Fig. 2C and D).

10.1128/mBio.01839-19.1 FIG S1 IFI44 mRNA expression is induced after virus infection and poly(I·C) and IFN treatments. (A) Human A549 cells were infected with influenza PR8 virus for 24 h, with r3LCMV-GFP/Gluc for 42 h, with rVSV-GFP for 24 h, and with SeV for 24 h. (B) Alternatively, A549 cells were treated with poly(I·C) or IFN-α for 16 h. Total RNAs were extracted, and the expression levels of IFI44 mRNA were evaluated by RT-qPCR. Error bars represent SDs of results of measurements performed in triplicate wells. Three different experiments were performed with similar results. *, *P* < 0.05 (using Student’s *t* test). Download FIG S1, PDF file, 0.01 MB. Copyright © 2019 DeDiego et al. 2019 DeDiego et al. This content is distributed under the terms of the Creative Commons Attribution 4.0 International license.

To confirm these results, human 293T cells were transiently transfected with a pCAGGS plasmid expressing IFI44 or with empty pCAGGS plasmid as an internal control. At 36 hpi, HA-tagged IFI44 expression was confirmed (data not shown). Next, 293T cells were infected with PR8 for 12 h, and expression levels of IFIT2 were evaluated by RT-qPCR. Interestingly, IFIT2 was induced to significantly lower levels in the cells overexpressing IFI44 than in the cells transfected with the empty plasmid (Fig. 3B), further suggesting that IFI44 expression negatively modulates the host antiviral responses induced after IAV infection.

To investigate IFI44 functions in the absence of virus replication, which might have been affecting the results, and to investigate whether IFI44 modulated IFN production or IFN signaling or both, IFI44 expression was knocked down in A549 cells and cells were transfected with poly(I·C), an analog of double-stranded RNA (dsRNA) mainly detected by the cytoplasmic PRRs RIG-I/MDA-5 (30), and 16 h later, IFN-β expression levels were analyzed by RT-qPCR (Fig. 3C). Alternatively, to evaluate the role of IFI44 in IFN signaling, A549 cells were treated with 250 U/ml of universal IFN-α, and IFIT2 expression levels were analyzed by RT-qPCR (Fig. 3D). As expected, IFI44 mRNA expression levels were induced after poly(I·C) and IFN-α treatments (Fig. S1B). The expression levels of IFN-β (Fig. 3C) and IFIT2 (Fig. 3D) were found to have increased 50-fold and 10-fold in the NT-silenced cells treated with poly(I·C) and IFN, respectively, compared to the nontreated cells, suggesting that these treatments induced strong antiviral responses. Interestingly, the levels of expression of IFN-β and IFIT2 were 2-fold and 8-fold higher in IFI44-silenced cells treated with poly(I·C) and IFN, respectively, than in the NT-transfected cells, suggesting that IFI44 silencing decreases IFN production and downregulates IFN signaling.

To analyze the effect of IFI44 in conferring biologically relevant IFN-mediated antiviral activity to virus infection, we assessed the effect of IFI44 downregulation (siRNA transfection) or overexpression (plasmid transfection) on viral infection (Fig. 3E and F). Human A549 cells were transfected with two different IFI44-siRNAs or with the pCAGGS plasmid expressing IFI44. At 36 h posttranscription (hpt), cells were transfected with poly(I·C) or treated with exogenous IFN-α to induce an antiviral state and were then infected with rVSV-GFP (MOI of 0.1), which is sensitive to the antiviral state induced by poly(I·C) and IFN treatments (25, 31). Then, rVSV-GFP production was analyzed at 24 hpi. In mock-treated cells, rVSV-GFP grew with high titers (>10^7^ PFU/ml) (Fig. 3E and F). In contrast, virus titers were decreased more than 250-fold in poly(I·C)-transfected and IFN-α-treated cells, consistent with the induction of a host antiviral state in these cells (Fig. 3E and F). Interestingly, in IFI44-silenced cells transfected with poly(I·C) or treated with IFN, the rVSV-GFP titers were at least 5-fold lower than the titers in NT siRNA-transfected cells (Fig. 3E). In contrast, rVSV-GFP titers were 3-fold higher in cells overexpressing IFI44 than in cells transfected with empty plasmid (Fig. 3F). These results correlate with our previous data (Fig. 3A to D), further demonstrating that IFI44 expression decreases the induction of host antiviral responses.

To analyze whether the effect of IFI44 on antiviral responses also applies to other species, IFI44 expression was silenced in mouse fibroblast L929 cells and cells were transfected with poly(I·C) to induce an antiviral state. Then, cells were infected (MOI of 0.1) with rVSV-GFP, and viral titers were analyzed at 24 hpi. IFI44 expression was induced after poly(I·C) treatment in NT siRNA-transfected cells (Fig. S2A) and was effectively knocked down in mock-treated and poly(I·C)-treated cells transfected with the IFI44 siRNA, as quantified at the mRNA level by quantitative PCR (qPCR) (Fig. S2A). No significant effect was observed on rVSV-GFP production in mock-treated cells when comparing cells silenced for IFI44 to control cells transfected with the NT siRNA. Importantly, in poly(I·C)-treated, NT siRNA-transfected cells, rVSV-GFP titers were found to have decreased around 500-fold compared to the results seen with the nontreated cells, consistent with the induction of an antiviral state mediated by poly(I·C) treatment. Interestingly, rVSV-GFP titers in poly(I·C)-treated, IFI44-siRNA-transfected cells were around 12-fold lower than in poly(I·C)-treated, NT siRNA-transfected cells (Fig. S2C). Furthermore, IFIT2 expression was increased in IFI44-silenced cells compared to NT siRNA-transfected cells (Fig. S2B). These results indicate that the effect of IFI44 with respect to negative modulation of innate immune responses applies to mice in addition to humans.

10.1128/mBio.01839-19.2 FIG S2 IFI44 negatively affects the antiviral state induced by IFN in mouse cells. Murine L929 cells were transfected with an IFI44-specific siRNA or with NT siRNA as an internal control. At 36 hpt, cells were transfected with poly(I·C) to induce an antiviral state or were left untreated (Mock) as a control. (A and B) Total RNAs were extracted, and the expression levels of IFI44 (A) and IFIT2 (B) mRNAs were evaluated by RT-qPCR. Error bars represent the SDs of results of measurements performed in triplicate wells. (C) Virus production in L929 cells infected with rVSV-GFP at an MOI of 0.1 was analyzed at 24 h postinfection (hpi). Bars represent SDs determined using duplicate wells. Three different experiments were performed, with similar results. *, *P* < 0.05 (for comparisons between NT- and IFI44-silenced cells performed using Student’s *t* test). Download FIG S2, PDF file, 0.02 MB. Copyright © 2019 DeDiego et al. 2019 DeDiego et al. This content is distributed under the terms of the Creative Commons Attribution 4.0 International license.

### KO IFI44 expression negatively modulates antiviral responses.

To confirm the data from our silencing and overexpression experiments showing that IFI44 expression negatively affects IFN responses, we obtained IFI44 knockout (KO) HAP-1 cells, using CRISPR/Cas9 technologies. To analyze whether IFI44 expression is induced by poly(I·C) and type I IFN in this IFI44 KO cell line, HAP-1 wild-type (WT) cells were transfected with poly(I·C) or treated with IFN-α and IFI44 mRNA and protein levels were evaluated by RT-qPCR (Fig. S3A) and Western blotting (Fig. S3B), respectively. IFI44 mRNA was induced by more than 300-fold and 4,000-fold in poly(I·C)-treated and IFN-treated HAP-1 WT cells, respectively, compared to mock-treated HAP-1 WT cells (Fig. S3A), indicating that IFI44 was induced by IFN-α in the HAP-1 WT cells. Correlating with the IFI44 mRNA levels, IFI44 protein was detected by Western blotting in the HAP-1 WT cells treated with two different concentrations (200 and 2,000 U/ML) of IFN-α, in comparison to the results seen with nontreated cells, in which the protein was not detected (Fig. S3B). As expected, in the HAP-1 IFI44 KO cells, IFI44 protein was not detected (Fig. S3B).

10.1128/mBio.01839-19.3 FIG S3 IFI44 expression is induced by IFN treatment in HAP-1 cells. HAP-1 WT and IFI44 KO cells were treated with poly(I·C) (2,000 ng/ml) or with IFN-α (panel A, 2,000 U/ml; panel B, 200 and 2,000 U/ml) or were left untreated (Mock) as a control. (A) Total RNAs were extracted, and the expression levels of IFI44 (A) mRNAs were evaluated by RT-qPCR. Error bars represent the SDs of results of measurements performed in triplicate wells. *, *P* < 0.05 (for comparisons between treated and nontreated cells performed using Student’s *t* test). (B) Western blotting was performed using anti-IFI44 (top) and actin (bottom) antibodies. Molecular weight markers are indicated (in kilodaltons) on the right. Download FIG S3, PDF file, 0.5 MB. Copyright © 2019 DeDiego et al. 2019 DeDiego et al. This content is distributed under the terms of the Creative Commons Attribution 4.0 International license.

**FIG 4:**
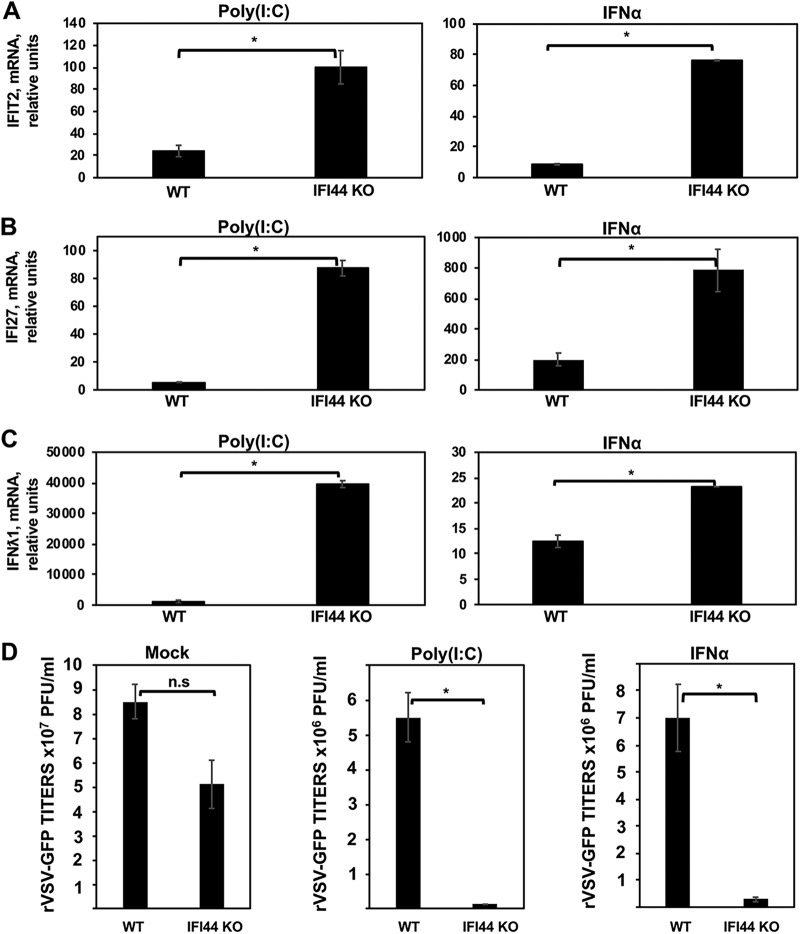
IFI44 protein impairs antiviral responses. HAP-1 WT and IFI44 KO cells were transfected with poly(I·C) or treated with IFN-α for 16 h. (A) Total cellular RNA was purified, and the levels of IFIT2 (A), IFI27 (B), and IFN-λ1 (C) mRNAs were evaluated by RT-qPCR. Bars represent SDs determined using duplicate wells. Three different experiments were performed with similar results. (D) Cells that had been subjected to mock treatment, transfected with poly(I·C), or treated with IFN-α were infected with rVSV-GFP (MOI of 0.1). Virus production was analyzed at 24 hpi. Bars represent SDs determined using duplicate wells. Three different experiments were performed, with similar results. *, *P* < 0.05 (for comparisons between HAP-1 WT and IFI44 KO cells).

To confirm that IFI44 negatively regulates IFN responses, WT and IFI44 KO HAP-1 cells were treated with IFN-α and poly(I·C) to induce IFN responses. Then, the levels of ISG IFIT2, ISG IFI27, and IFN-λ1 mRNAs were evaluated by qPCR (Fig. 4A to C, respectively). IFN-λ1 is a type III IFN and is induced by viruses and IFNs, such as IFN-α (32). As expected, expression of IFIT2, IFI27, and IFN-λ1 was induced after IFN-α and poly(I·C) treatments (Fig. 4A to C). Interestingly, the expression levels of IFIT2 were 4-fold and 8-fold higher in IFI44-KO cells treated with poly(I·C) and IFN-α, respectively, than in WT cells (Fig. 4A). Similarly, the expression levels of IFI27 were 15-fold and 4-fold higher in IFI44 KO cells treated with poly(I·C) and IFN-α, respectively, than in WT cells (Fig. 4B). Furthermore, the levels of IFN-λ1 mRNAs were 40-fold and 2-fold higher in IFI44-KO cells treated with poly(I·C) and IFN-α, respectively, than in WT cells (Fig. 4C), further demonstrating that IFI44 negatively affects IFN responses.

To study the effect of IFI44 in the induction of antiviral states, WT and IFI44 KO HAP-1 cells treated with poly(I·C) and IFN-α were infected with rVSV-GFP (MOI of 0.1) and virus production was analyzed at 24 hpi. In mock-treated cells, rVSV-GFP grew with high titers (>10^7^ PFU/ml) (Fig. 4D). In contrast, rVSV-GFP titers were decreased more than 20-fold in poly(I·C)-transfected and IFN-α-treated cells, as expected and consistent with the induction of a host antiviral state (Fig. 4D). Correlating with the RT-qPCR results from IFIT2 and IFN-λ1 genes showing antiviral activity (3, 33), rVSV-GFP titers in IFI44 KO cells transfected with poly(I·C) or treated with IFN-α were 100-fold and 20-fold lower than those observed in parental HAP-1 WT cells (Fig. 4D). Moreover, these data correlate with our previous findings obtained using knockdown or overexpression of IFI44 (Fig. 3; see also Fig. S2C).

Human HAP-1 cells are myeloid cells, in contrast to human A549 cells, which are epithelial cells. To further analyze the effect of IFI44 on IFN responses in the HAP-1 cells, parental HAP-1 WT cells were silenced with NT or IFI44 siRNAs and treated with poly(I·C), and the levels of induction of IFI44, IFIT2, and IFN-λ1 were analyzed by RT-qPCR (Fig. S4A to C). IFI44 mRNA levels were decreased around 6-fold in IFI44 siRNA-transfected cells compared to NT siRNA-transfected cells (Fig. S4A). As observed with A549 cells, IFI44 silencing in HAP-1 cells increased IFIT2 and IFN-λ1 induction after poly(I·C) transfection (Fig. S4B and C). Correlating with these results, rVSV-GFP titers in poly(I·C)-transfected cells were around 10-fold lower in cells transfected with the IFI44 siRNA than in the NT siRNA-transfected cells (Fig. S4D). These results confirm that IFI44 expression decreased the induction of host antiviral responses as indicated by the results using IFI44 KO cells and cells in which the level of expression of IFI44 was specifically knocked down using siRNAs.

10.1128/mBio.01839-19.4 FIG S4 IFI44 impairs IFN responses in myeloid tissue-derived cells. Human HAP-1 control cells were transfected with an IFI44-specific siRNA or with NT siRNA as an internal control. At 36 hpt, cells were transfected with poly(I·C) to induce an antiviral state or were left untreated (Mock) as a control. (A to C) Total RNAs were extracted, and the expression levels of IFI44 (A), IFIT2 (B), and IFNL1 (C) mRNAs were evaluated by RT-qPCR. Error bars represent the SDs of results of measurements performed in triplicate wells. (D) Virus production in HAP-1 control cells infected with rVSV-GFP at an MOI of 0.1 was analyzed at 24 hpi. Bars represent SDs determined using duplicate wells. Three different experiments were performed, with similar results. *, *P* < 0.05 (for comparisons between NT- and IFI44-silenced cells performed using Student’s *t* test). Download FIG S4, PDF file, 0.02 MB. Copyright © 2019 DeDiego et al. 2019 DeDiego et al. This content is distributed under the terms of the Creative Commons Attribution 4.0 International license.

### IFI44 interacts and colocalizes with FKBP5.

Since the role of IFI44 in regulating innate immune responses has not been described previously, we analyzed host cell proteins binding to IFI44. To that end, human 293T cells were transiently transfected with a plasmid expressing IFI44-HA or with plasmids expressing HA-tagged irrelevant proteins (IFI6 and IFI27) as negative controls. Next, IFI44-, IFI6-, and IFI27-associated complexes were purified by the use of anti-HA affinity columns and eluted. Mass spectrometry analysis demonstrated that in cells overexpressing IFI44-HA, IFI44 was immunoprecipitated with a high (90%) level of coverage and with 39 distinct peptides identified, whereas this protein was not detected in cells transfected with the empty plasmid or in the cells transfected with plasmids expressing IFI6-HA and IFI27-HA proteins that were used as controls. The second most highly represented protein in the samples, which was specifically associated with IFI44-HA, but not with IFI6-HA and IFI27-HA, was endogenous FKBP5. FKBP5 was detected with a level of coverage higher than 57%, and 24 peptides were identified.

**FIG 5:**
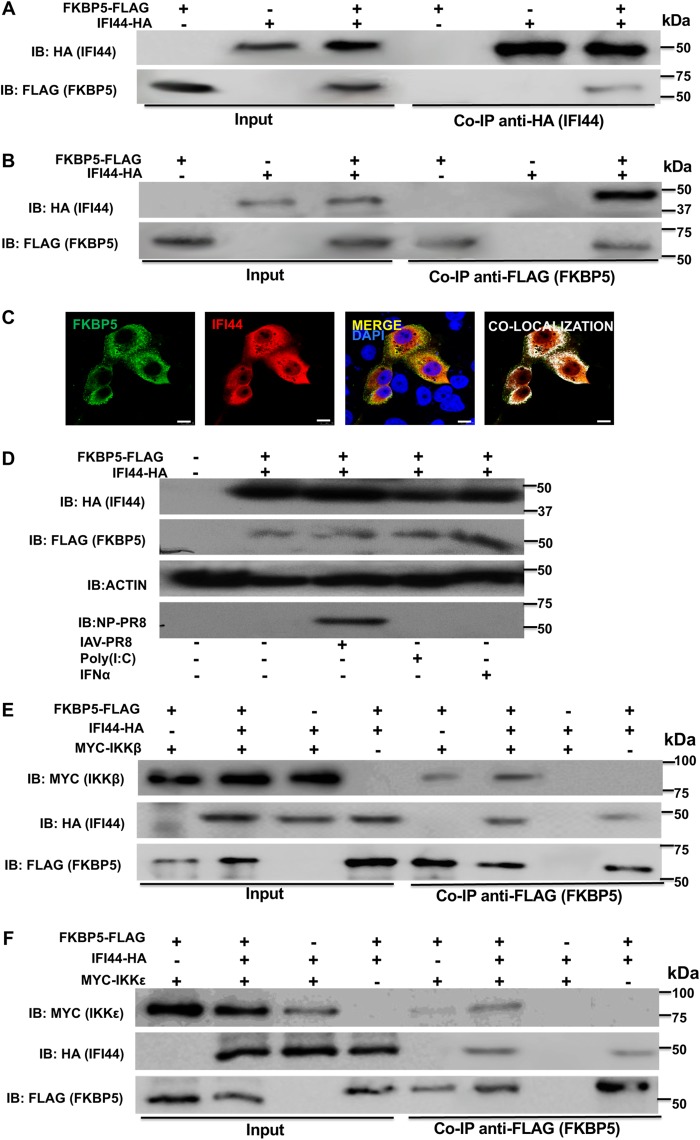
IFI44 interacts with FKBP5 and does not inhibit binding of FKBP5 to IKKβ or IKKε. (A to C) Human 293T cells were transiently cotransfected with the pCAGGS plasmid encoding IFI44-HA and FKBP5-FLAG, or with empty plasmids, as internal controls. IB, immunoblot. (A and B) Coimmunoprecipitation experiments using anti-HA to pull down IFI44 (A) and anti-FLAG to pull down FKBP5 (B) using affinity columns were performed. Western blotting using antibodies specific for the HA tag (to detect IFI44) or the FLAG tag (to detect FKBP5 protein) was performed to detect protein in the cellular lysates (Input) and after the Co-IP. Molecular weight markers are indicated (in kilodaltons) on the right. Three different experiments were performed, with similar results. (C) At 24 hpi, cells were fixed with paraformaldehyde, FKBP5-FLAG and IFI44-HA were labeled with antibodies specific for the tags (in green and red, respectively), and nuclei were stained with DAPI (in blue). Areas of colocalization of both proteins appear in yellow in the third picture and in white in the fourth picture. Scale bar, 10 μm. (D) Human A549 cells were transiently cotransfected with the pCAGGS plasmid encoding IFI44-HA and FKBP5-FLAG, or with empty plasmids, as internal controls. At 24 hpt, the cells were subjected to mock treatment, transfected with poly(I·C), treated with IFN-α, or infected with IAV for 24 h. Western blotting using antibodies specific for the HA tag (to detect IFI44), the FLAG tag (to detect FKBP5), anti-actin, and IAV anti-NP proteins was performed. Molecular weight markers are indicated (in kilodaltons) on the right. Two different experiments were performed, with similar results. (E and F) Human 293T cells were transiently cotransfected with different combinations of pCAGGS plasmids encoding IFI44-HA, FKBP5-FLAG, and MYC-IKKβ (E) or IFI44-HA, FKBP5-FLAG, and MYC-IKKε (F). Co-IP experiments using an anti-FLAG affinity column (to pull down FKBP5) were performed. Western blotting using antibodies specific for the MYC tag (to detect IKKβ or IKKε), the HA tag (to detect IFI44), and the FLAG tag (to detect FKBP5) was performed to detect protein in the cellular lysates (Input) and after the Co-IP. Molecular weight markers are indicated (in kilodaltons) on the right. Three different experiments were performed, with similar results.

To confirm that IFI44 interacts with FKBP5, a pCAGGS plasmid encoding FKBP5 fused to a FLAG epitope tag was transiently cotransfected in 293T cells together with the pCAGGS plasmid encoding IFI44-HA, or with pCAGGS empty plasmid as a control, and Co-IP experiments using an anti-HA antibody (to pull down IFI44; Fig. 5A) and an anti-FLAG antibody (to pull down FKBP5; Fig. 5B) were performed. Western blotting revealed that IFI44 specifically coimmunoprecipitated with FKBP5 by the use of affinity columns coupled to anti-HA and anti-FLAG antibodies (Fig. 5A and B, respectively), indicating that the two proteins interacted in the cell. Moreover, to confirm that the two proteins interacted during viral infection, 293T cells were cotransfected with pCAGGS-IFI44-HA and pCAGGS-FKBP5-FLAG plasmids, and the cells were infected with IAV at 24 hpt for an additional 24 h. Similarly to noninfected cells, analysis performed using anti-HA antibodies showed that the two proteins coimmunoprecipitated together (data not shown), indicating that they are able to interact during IAV infection.

To analyze whether IFI44 and FKBP5 colocalize intracellularly, cells were transiently transfected with pCAGGS plasmids expressing FKBP5 and IFI44 proteins and the localization of both proteins was analyzed by immunofluorescence and confocal microscopy (Fig. 5C). IFI44 expression was detected in the cytoplasm and in the nucleus, as previously described (34). In contrast, FKBP5 was detected mainly in the cytoplasm (Fig. 5C). Importantly, colocalization of IFI44 and FKBP5 was observed in distal regions of the cytoplasm (Fig. 5C), reinforcing the conclusions from the Co-IP experiments and further demonstrating that IFI44 and FKBP5 interact inside the cell (Fig. 5A and B).

Importantly, to analyze whether IAV infection, poly(I·C) transfection, and IFN-α treatment affect the expression of IFI44 and FKBP5 at the protein level, A549 cells were cotransfected with plasmids expressing IFI44-HA and FKBP5-FLAG, and at 24 hpt, the cells were subjected to mock treatment, transfected with poly(I·C), treated with IFN-α, or infected with IAV for 24 h. Then, the expression of IFI44-HA, FKBP5-FLAG, actin (as a control), and the viral nucleoprotein (NP) (as a marker of viral infection) was analyzed by Western blotting. These conditions did not significantly affect the expression of IFI44-HA or FKBP5-FLAG (Fig. 5D). Correlating with these results, the levels of FKBP5 mRNA, measured by RT-qPCR, were not significantly affected after poly(I·C) and IFN treatments (data not shown).

It has been previously shown that FKBP5 interacts with IKKα, IKKβ, and IKKγ and facilitates IKK complex assembly, leading to increased IKKα and IKKβ kinase activity, NF-κB activation, and IFN production (11). Furthermore, FKBP5 interacts with TRAF6 and TRAF3 and induces the expression of IFN and proinflammatory cytokines (35). In addition, it has been suggested that FKBP5 interacts with IKKε, likely increasing IKKε kinase activity (12) and, therefore, production and signaling of IFN (7, 14). To analyze whether the interaction of IFI44 with FKBP5 disrupts the interaction of FKBP5 with these kinases, human 293T cells were transiently cotransfected with pCAGGS plasmids expressing FKBP5-FLAG and MYC-IKKβ (Fig. 5E) or expressing FKBP5-FLAG and MYC-IKKε (Fig. 5F) in the presence or absence of the pCAGGS plasmid expressing IFI44-HA. FKBP5 specifically interacted with IKKβ (Fig. 5E) and IKKε (Fig. 5F), confirming previous reports (11, 12). However, these interactions were not abolished in the presence of IFI44 (Fig. 5E and F), suggesting that the binding of IFI44 to FKBP5 does not inhibit its binding to IKKβ and to IKKε.

### IFI44 negatively affects the kinase activity of IKKβ and IKKε.

**FIG 6:**
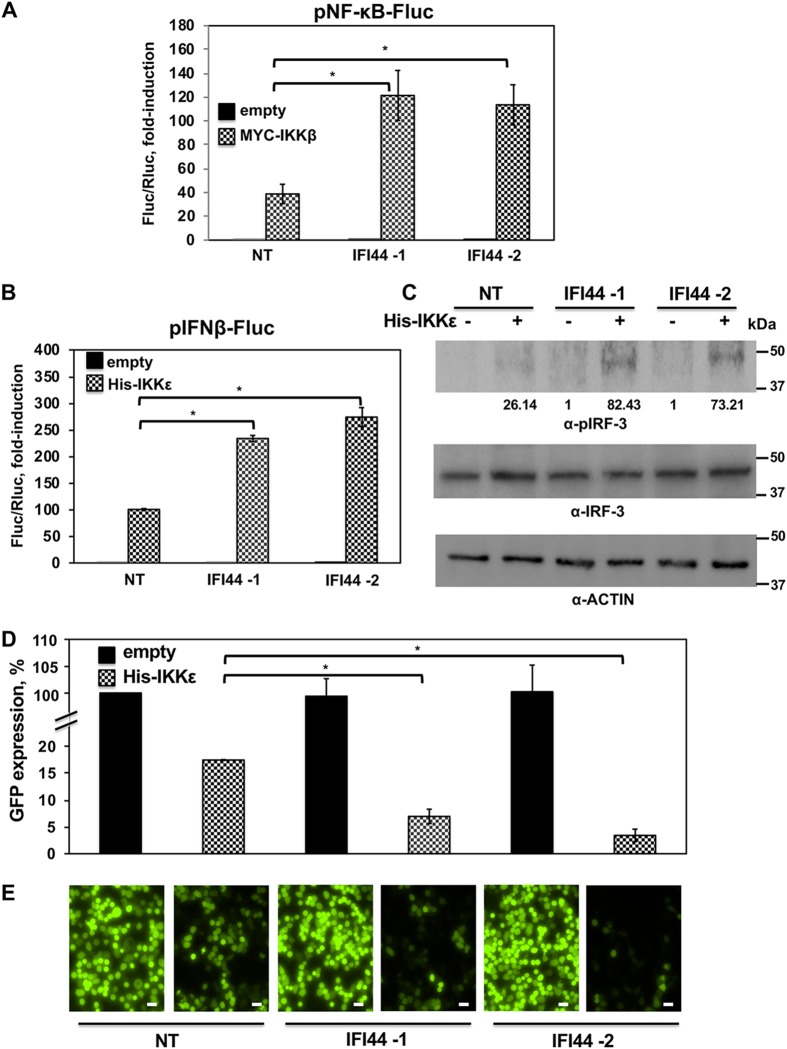
IFI44 negatively affects IKKβ and IKKε activation. (A) IFI44-silenced human 293T cells were transiently cotransfected with a plasmid expressing IKKβ, a plasmid expressing the Fluc reporter gene under the control of NF-κB (pNF-κB-Fluc), and a plasmid constitutively expressing Rluc. (B) IFI44-silenced human 293T cells were transiently cotransfected with a plasmid expressing IKKε together with a plasmid expressing the Fluc reporter gene under the control of IFN-β promoter (pIFNβ-Fluc) and a plasmid constitutively expressing Rluc. (A and B) At 24 hpt, levels of Fluc were determined and normalized to the levels of Rluc. Data represents means and SDs of results from triplicate wells. Experiments were repeated three times with similar results. (C) Cellular lysates from cells analyzed as described for panel B were collected, and protein levels of pIRF-3 and actin were evaluated by Western blotting. Western blots were quantified by densitometry using ImageJ software (v1.46), and the amounts of pIRF-3 were normalized to the amounts of actin (numbers below the pIRF-3 blot). Molecular weight markers are indicated (in kilodaltons) at the right. (D and E) Tissue culture supernatants from cells analyzed as described for panel B were collected and used to treat fresh A549 cells. After 24 h of incubation, cells were infected (MOI of 0.1) with rVSV-GFP. At 24 hpi, GFP expression was quantified in a microplate reader (D) and GFP-infected cells were analyzed by visualizing GFP expression under a fluorescence microscope (E). Experiments were repeated three times with similar results. *, *P* < 0.05 (using Student’s *t* test [panels A, B, and D]). Representative images from a microscope using a 20× objective are shown in panel E. Scale bars, 50 μm.

It has been previously shown that FKBP5 knockdown attenuates IKKα and IKKβ catalytic activity, leading to reduced NF-κB activation (11). This is because IKKα and IKKβ phosphorylate inhibitory molecules, including IkBα, which leads to NF-κB activation (36). To analyze whether IFI44 modulates IKKβ activity, human 293T cells were silenced with two different IFI44 siRNAs and cotransfected with a plasmid expressing Fluc under the control of the NF-κB promoter and a plasmid expressing Rluc under the control of a constitutively promoter to normalize the levels of transfection, together with a pCAGGS plasmid expressing MYC-IKKβ, or the empty pCAGGS plasmid as a control. At 24 h posttransfection, the levels of Fluc were measured and normalized to the levels of Rluc. As expected, MYC-IKKβ overexpression induced NF-κB activation (Fig. 6A) (9). NF-κB was activated to (around 2.5-fold) higher levels in IFI44-silenced cells than in NT siRNA-transfected cells (Fig. 6A), strongly suggesting that IFI44 modulates IKKβ activity.

In addition to its modulating IKKβ activity, it has been proposed that the binding of FKBP5 to IKKε may also modulate the kinase activity of IKKε (12). IKKε phosphorylates transcription factors IRF-3 and IRF-7, leading to type I IFN induction (7). In addition, IKKε phosphorylates STAT1 for IFN-inducible antiviral responses (14, 15). To analyze whether IFI44 modulates IKKε activity, cells silenced for IFI44 with two different siRNAs were transiently cotransfected with a plasmid expressing Fluc under the control of an IFN-β-driven promoter, a plasmid expressing the Rluc constitutively (to normalize the levels of transfection), and a pCAGGS plasmid expressing His-IKKε or an empty plasmid as a control. Levels of Fluc driven by the IFN-β promoter were 3-fold higher in the IFI44-silenced cells than in the NT-silenced cells (Fig. 6B), strongly suggesting that IFI44 negatively regulates IFN production mediated by IKKε activation. On the basis of these results, we evaluated the levels of phosphorylated IRF-3 (pIRF-3) under the same experimental conditions (Fig. 6C). As expected, IKKε overexpression resulted in IRF-3 phosphorylation (Fig. 6C). Interestingly, levels of pIRF-3 were higher in cells silenced for IFI44 than in cells transfected with the NT control siRNA (Fig. 6C), demonstrating that IFI44 also negatively modulates IKKε activity.

To further confirm these results, a well-established virus-based IFN bioassay was performed (25). Tissue culture supernatants from the experiment shown in Fig. 6B were used to treat fresh A549 cells for 24 h. Then, cells were infected with rVSV-GFP, and at 24 hpi, the levels of GFP were quantified using a microplate reader (Fig. 6D) and fluorescence was observed under a fluorescence microscope (Fig. 6E). As expected, rVSV-GFP replication, as determined by GFP expression, was lower in cells treated with tissue culture supernatants from IKKε-transfected cells than in cells treated with empty-plasmid-transfected cell supernatants (Fig. 6D and E), consistent with the idea of IFN production in cells overexpressing IKKε. Interestingly, the levels of rVSV-mediated GFP expression were lower in the cells treated with the supernatants coming from pCAGGS-IKKε-transfected cells which were silenced with the two IFI44 siRNAs than in the cells treated with the supernatants coming from pCAGGS-IKKε-transfected cells which were silenced with the NT control siRNA (Fig. 6D and E). These data correlate with our findings that IFI44 silencing increases IFN responses after IKKε overexpression (Fig. 6B).

**FIG 7:**
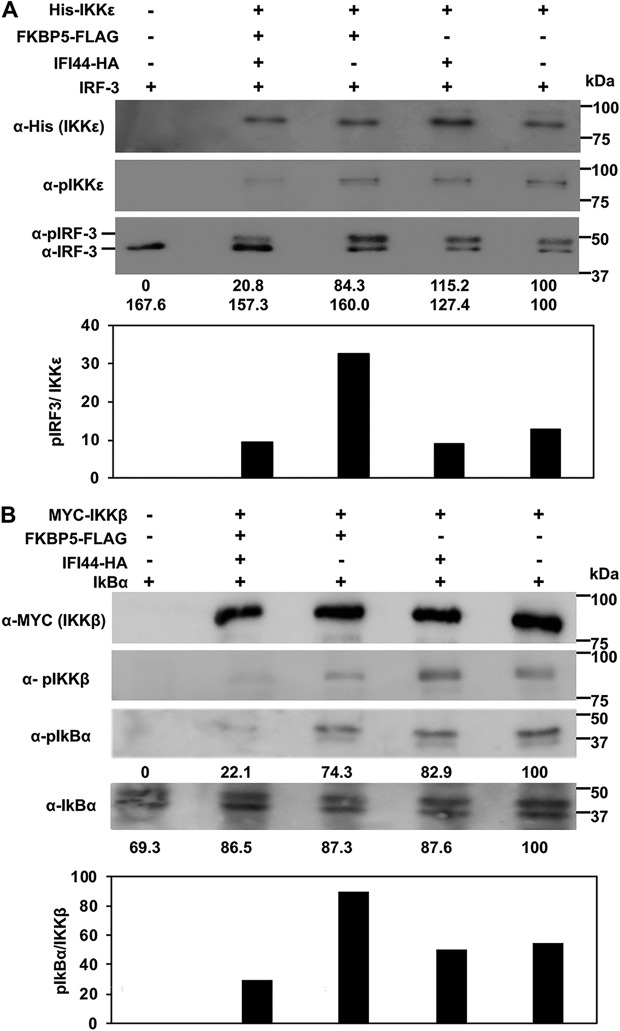
IFI44 decreases the kinase activity of IKKβ and IKKε. Human 293T cells were silenced for IFI44, or for FKBP5, and were transfected with plasmids expressing His-IKKε (A) or MYC-IKKβ (B), together with IFI44-HA, and FKBP5-FLAG expression plasmids. At 24 hpt, IKKε (A) and IKKβ (B) complexes were purified with anti-His and anti-MYC antibodies, respectively, and these complexes were assayed in kinase assays using IRF-3 (for the IKKε complexes shown in panel A) and IkBα (for the IKKβ complexes shown in panel B) as substrates. The levels of phosphorylated and unphosphorylated forms of IRF-3 (panel A, bottom blot) and IkBα (panel B, third and fourth blots) were analyzed by Western blotting using specific antibodies. Levels of IKKε were analyzed using an anti-His-specific antibody (A, first blot) and anti-pIKKε (A, second blot), and levels of IKKβ were analyzed using an anti-MYC-specific antibody (B, first blot) and anti-pIKKβ (B, second blot). Western blots were quantified by densitometry using ImageJ software (v1.46). Protein expression levels in cells expressing IKKε (A) and IKKβ (B) alone were assigned a value of 100% for comparisons with the levels of expression in cells expressing the different combinations of IKKε/IFI44/FKBP5 (A) or IKKβ/IFI44/FKBP5 (B) (numbers are indicated below each plot). pIRF-3 and IRF-3 levels (observed in the same bottom blot in panel A) and pIkBα and IkBα (third and bottom blot in panel B) are represented with numbers below each blot. Levels of pIRF-3 and pIkBα normalized to the levels of IKKε and IKKβ are represented in the bottom graphs in panels A and B, respectively. Molecular weight markers are indicated (in kilodaltons) on the right.

It has been reported previously that signaling pathways leading to type I IFN production involve the phosphorylation of IRF-3 by IKKε (7) and the phosphorylation of IkBα by IKKβ (36). IFI44 did not show any ability to phosphorylate IRF-3 and IkBα in kinase assays (data not shown). To analyze whether IFI44 negatively regulates IKKβ and IKKε activity and whether any such regulation was dependent on FKBP5 expression, cells were silenced for IFI44 or for FKBP5 and were then transiently transfected with pCAGGS plasmids expressing His-IKKε or MYC-IKKβ, IFI44-HA, and FKBP5-FLAG. At 24 hpt, IKKε and IKKβ complexes were purified with anti-His and anti-MYC antibodies, respectively, and these complexes were assayed in kinase assays using purified IRF-3 (for IKKε complexes) and purified IkBα (for IKKβ complexes) as substrates. Expression of IKKε and IKKβ (Fig. 7A and B, respectively) and of FKBP5 and IFI44 (data not shown) was confirmed in all the experiments. Silencing of FKBP5 was confirmed at the mRNA level (Fig. S5A) and at the protein level (Fig. S5B). Total and phosphorylated levels of IRF-3, IKKε, IkBα, and IKKβ were analyzed by Western blotting using specific antibodies (Fig. 7A and B, respectively). pIRF-3 and pIkBα were observed when IKKε and IKKβ were expressed, in contrast to the cells transfected with an empty plasmid control, indicating that IKKε and IKKβ were responsible for IRF-3 and IkBα phosphorylation, respectively, in those assays. Interestingly, when IFI44 was expressed together with FKBP5 and IKKε or IKKβ, the levels of pIRF-3 and pIkBα (normalized to the levels of IKKε and IKKβ present in the complexes) were 3-fold to 4-fold lower than when IFI44 was not expressed. However, the levels of nonphosphorylated IRF-3 and IkBα were similar in both cases (Fig. 7A and B, lower panels). Interestingly, when FKBP5 was not present in the complexes, the presence or absence of IFI44 had no effect on the levels of IRF-3 and IkBα phosphorylation (Fig. 7A and B, lower panels). Importantly, expression of IFI44 did not significantly affect the expression levels of IKKε and IKKβ (Fig. 7A and B, respectively, top panels) but did affect the levels of phosphorylated (phospho-) IKKε and phospho-IKKβ (Fig. 7A and B, respectively, middle panels), measured as hallmarks of activation (37, 38). These results suggest that IFI44 negatively affects IKKβ and IKKε kinase activities and that this effect is likely dependent on the binding of IFI44 to FKBP5. This negative outcome of the effect of IFI44 on IKKβ and IKKε activities most likely represents the mechanism used by IFI44 to regulate IFN responses.

10.1128/mBio.01839-19.5 FIG S5 FKBP5 mRNA and protein expression levels are effectively knocked down by siRNAs. Human 293T cells were transfected with NT- or FKBP5-specific siRNAs for 36 h. (A) Total RNAs were extracted, and expression of FKBP5 mRNA was evaluated by RT-qPCR. Error bars represent the SDs of results of measurements performed in triplicate wells. *, *P* < 0.05 (for comparisons between NT- and FKBP5-silenced cells performed using Student’s *t* test). (B) At 36 h after siRNA transfection, cells were transfected with a plasmid expressing FKBP5-FLAG for 48 h. A Western blot analysis using an anti-FLAG-specific antibody (to detect FKBP5) and anti-actin antibody (internal control) was performed. Protein expression levels in cells silenced with the NT siRNA were assigned a value of 100% for comparisons with the levels of expression in IFI44-silenced cells (numbers below the HA blot). FKBP5 expression was normalized to actin expression. Molecular weight markers are indicated (in kilodaltons) on the right. Three different experiments were performed, with similar results. Download FIG S5, PDF file, 0.04 MB. Copyright © 2019 DeDiego et al. 2019 DeDiego et al. This content is distributed under the terms of the Creative Commons Attribution 4.0 International license.

## DISCUSSION

**FIG 8:**
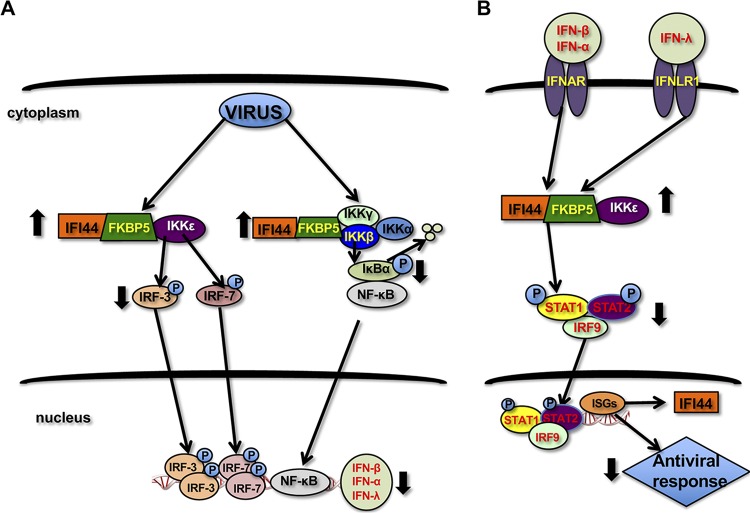
Graphical abstract of the effects of IFI44 on IFN responses. After virus infections, viral components are recognized by PRRs. This recognition leads to the activation of kinases such as IKKε, which phosphorylates and activates transcription factors IRF-3 and IRF-7, and IKKα and IKKβ, which phosphorylates IκBα, leading to IκBα degradation and NF-κB activation. IRF-3, IRF-7, and NF-κB are critical for the induction of expression of type I and III IFNs. Type I and III IFNs signal through IFNAR and IFNLR1, respectively, leading to STAT1 and STAT2 phosphorylation. In addition, STAT1 is phosphorylated by IKKε during IFN signaling, which is the step critical for the induction of IFN antiviral responses. Activation of STAT1/STAT2 leads to the induction of ISGs with antiviral activities, including IFI44. IFI44 interacts with FKBP5, which in turn interacts with IKKα, IKKβ, and IKKε kinases. In the presence of FKBP5, IFI44 decreases the ability of IKKε to phosphorylate IRF-3 (and, most probably, IRF-7 and STAT1) and the ability of IKKβ to phosphorylate IkBα. As a consequence, the levels of IRF-3 activation, NF-κB activation, and IFN production (A) are decreased in the presence of IFI44, leading to diminished antiviral responses (B) after virus infections.

In this work, we describe a completely novel role for IFI44 in negatively regulating innate immune responses induced by infection with different viruses, including IAV, VSV, SeV, and LCMV (Fig. 8). After virus infections, type I and III IFNs are produced, through a mechanism involving the recognition of virus components by PRRs such as RIG-I, MDA-5, and TLRs (6). This recognition leads to the activation of kinases such as IKKε, which phosphorylates and activates the transcription factors IRF-3 and IRF-7 (7), and the activation of IKKα and IKKβ, which phosphorylate IκBα, leading to IκBα degradation and NF-κB activation (10). IRF-3, IRF-7, and NF-κB are critical for the induction of expression of type I and III IFNs (Fig. 8) (1). Type I and III IFNs signal through the IFN-α/β receptor (IFNAR) and IFN-λ receptor (IFNλR1), respectively, leading to STAT1 and STAT2 phosphorylation and activation by the tyrosine kinases JAK1 and TYK2 (2) (Fig. 8). In addition, STAT1 is phosphorylated by IKKε during IFN signaling, this step being critical for the induction of IFN antiviral responses (14, 15) (Fig. 8). Activation of STAT1/STAT2 leads to the induction of ISGs with antiviral activities (2), including IFI44 (18, 19) (Fig. 8). In this work, we describe a completely novel interaction of IFI44 with FKBP5 (Fig. 5A to C), which in turn interacts with IKKα, IKKβ, and IKKε kinases (Fig. 5E and F) (11). In the presence of FKBP5, IFI44 decreases the ability of IKKε to phosphorylate IRF-3 (and most probably IRF-7 and STAT1) (Fig. 7A and data not shown) and the ability of IKKβ to phosphorylate IkBα (Fig. 7B). As a consequence, IFN production (Fig. 3C; see also Fig. 6B), IFN signaling (Fig. 3D), and NF-κB activation (Fig. 6A) are increased in the absence of IFI44, and therefore IFN responses are induced to a higher extent. Furthermore, we report that IFI44 silencing negatively affects virus production (Fig. 2C and D), most probably because of its effects in negatively modulating innate immune responses.

IFI44 is a type I IFN-induced protein (18, 19) and is upregulated after infection with different viruses, such as SeV, LCMV, VSV, and IAV (Fig. 1; see also Fig. S1A). In contrast to the general idea that ISGs display antiviral functions, here, we show novel data suggesting that knocking down the expression of IFI44 decreased viral production of different viruses such as IAV and LCMV (Fig. 2C and D) and that IFI44 negatively modulated IFN responses (Fig. 3; see also Fig. 4 and Fig. S2 and S4), even under conditions in which we did not observe a complete silencing of IFI44 (Fig. 1). This is not the first study reporting cellular factors, including ISGs, which negatively regulate IFN responses. For example, it has been shown that the ISGs IFI35 (39) and ISG56/IFIT1 (40); the ubiquitin ligases RING finger protein-5 (RNF-5) (41), RNF-125 (42), and RANBP2-type and C3HC4-type zinc finger containing 1 (RBCK1) (43); and other cellular factors such as suppressor of IKBKE (SIKE) (44) and A20 (45) negatively modulate innate immune responses, although the modulation occurs through mechanisms and targets different from those described in this study for IFI44. An excessive level of production of IFN and other cytokines could be deleterious to the host, and negative-feedback mechanisms such as those provided by these ISG proteins are needed.

The effect of IFI44 knockdown or knockout on virus production (Fig. 2C and D) is most probably a consequence of the negative effect of IFI44 on virus-induced innate immune responses (Fig. 3 and 4; see also Fig. S2 and S4). It is known that IAV and LCMV, like other viruses, are sensitive to the antiviral states induced by IFN (3, 27–29) and that many ISGs such as Myxovirus resistance protein 1 (MX1), IFN-induced transmembrane proteins (IFITM), oligoadenylate synthetase (OAS), protein kinase R (PKR), IFIT1, and IFIT2 restrict IAV replication (3). According to the potent effect of IFN in restricting virus replication, viruses encode proteins which counteract innate immune responses induced by the host, allowing the virus to replicate more efficiently in the cells, including such proteins as IAV nonstructural 1 (NS1) protein (46) and LCMV nucleoprotein (NP) (47). Accordingly, IAVs lacking the NS1 protein (48) or LCMVs affecting the anti-IFN function of the viral NP (47) induce a high-level IFN response and cannot replicate in IFN-competent systems.

The IFI44 overexpression/silencing/knockout experiments showed a modulation of poly(I·C)- and IFN-mediated inhibition of rVSV-GFP growth (Fig. 3E and F; see also Fig. 4 and Fig. S2C and S4D), correlating with the results showing that IFI44 negatively regulates IFN responses (Fig. 3; see also Fig. 4 and Fig. S2 and S4). Similarly, expression of the ISGs IFI35 (39), ISG56/IFIT1 (40), and RNF5 (41), which are negative-feedback regulators of virus-induced IFN responses, also reversed cytoplasmic poly(I·C)-induced inhibition of VSV replication.

In addition, we have shown for the first time that IFI44 interacts with the immunophilin FKBP5 (Fig. 5). Immunophilins comprise a family of chaperones that are members of a highly conserved family of proteins all of which are *cis*-*trans* peptidyl-prolyl isomerases (49, 50). FKBP5 binds IKKα, IKKβ, and IKKγ and facilitates IKK complex assembly, leading to increased IKKα and IKKβ kinase activity (11). The phosphorylation of IκB inhibitors mediated by IKKβ and IKKα leads to their degradation, allowing NF-κB to migrate to the nucleus and activate IFN and proinflammatory cytokine expression (10). In addition, it has been proposed that FKBP5 interacts with IKKε (12), an interaction that was confirmed in this work (Fig. 5F). IKKε phosphorylates the transcription factors IRF-3 and IRF-7 (7), leading to their activation and to induction of IFN (6). Considering that IFI44 interacts with FKBP5 (Fig. 5A to C) and that FKBP5 interacts with IKKα, IKKβ, and IKKε kinases (Fig. 5E and F) (11), we analyzed whether the interaction of IFI44 with FKBP5 affects the kinase activity of IKKβ and/or IKKε. Our data suggest that IFI44 does not disrupt the interaction of FKBP5 with IKKβ and IKKε (Fig. 5E and F). Notably, in the presence of FKBP5 (but not in its absence), the levels of pIκBα and pIRF-3 were lower in the presence of IFI44 (Fig. 7A and B), strongly supporting the results indicating that IFI44 negatively affects the kinase activities of IKKβ and IKKε and that, for this function, FKBP5 needs to be expressed. These data identify a novel mechanism for the negative-feedback modulation of IFN responses by IFI44.

Overall, we have described a new function for IFI44 in impairing host IFN responses. In addition, the results show that IFI44 silencing decreases virus production. Therefore, we propose that targeting IFI44 could be relevant for reducing virus-mediated diseases and also for controlling diseases associated with excessive immune signaling.

## MATERIALS AND METHODS

### Cells.

Madin-Darby canine kidney (MDCK) epithelial cells (ATCC CCL-34), human embryonic kidney cells (293T; ATCC CRL-11268), human lung epithelial carcinoma cells (A549; ATCC CCL-185), African green monkey kidney epithelial cells (Vero; ATCC CCL-81), and murine fibroblast cells (L929; ATCC CCL-1) were grown at 37°C in air enriched with 5% CO_2_ using Dulbecco’s modified Eagle’s medium (DMEM; Gibco) supplemented with 10% fetal bovine serum (Gibco) and with 50 μg/ml gentamicin (Gibco). Human HAP-1 haploid wild-type (WT) cells and HAP-1 IFI44 knockout (KO) cells produced using the CRISPR/Cas9 technology were obtained from Horizon Discovery, Inc. These cells were grown at 37°C in air enriched with 5% CO_2_ using Iscove’s modified Dulbecco's medium supplemented with 10% fetal bovine serum (Gibco) and with 50 μg/ml gentamicin (Gibco).

### Viruses.

Virus stocks of influenza A/Puerto Rico/8/1934 H1N1 (PR8) virus were grown in MDCK cells (51). Sendai virus (SeV), Cantell strain (52), was grown in embryonated chicken eggs. Recombinant vesicular stomatitis virus, Indiana strain, encoding green fluorescent protein (GFP) (rVSV-GFP) (53), and recombinant trisegmented LCMV, Armstrong strain, expressing GFP and Gaussia luciferase (r3LCMV-GFP/Gluc) (54, 55), were grown in Vero cells.

### Plasmids.

Polymerase II expression pCAGGS plasmids (56) encoding human IFI44 (GenBank accession number NM_006417.4) fused to an hemagglutinin (HA) epitope tag (pCAGGS-IFI44-HA); IFI6 and IFI27 (GenBank accession numbers NM_002038 and NM_001288952, respectively) fused to an HA tag (pCAGGS-IFI6-HA and pCAGGS-IFI27-HA); FKBP5 protein (GenBank accession number NM_004117.3) fused to a FLAG epitope tag (pCAGGS-FKBP5-FLAG); IKKβ and IKKε (GenBank accession numbers NM_001556 and NM_014002) fused to a MYC epitope tag (pCAGGS-MYC-IKKβ, and pCAGGS-MYC-IKKε, respectively); and IKKε fused to a 6×His tag (pCAGGS-His-IKKε) were generated by RT-PCR using total RNA isolated from human epithelial A549 cells and cloned using standard techniques (primers available upon request).

### Virus titrations.

The PR8 strain was titrated in MDCK cells by immunofocus assay (with results reported as fluorescent focus units [FFU] per milliliter), as previously described (57). All PR8 infections were performed in the presence of 1 μg/ml of tosylsulfonyl phenylalanyl chloromethyl ketone (TPCK)-treated trypsin (Sigma). r3LCMV-GFP/Gluc was titrated in Vero cells by fluorescence assay, as previously described (54, 55). rVSV-GFP was titrated by plaque assay (and quantified as PFU per milliliter) in Vero cells as previously described (25).

### siRNA-mediated silencing.

Human A549, 293T, or HAP-1 cells were transfected independently with two different “silencer select” small interfering RNAs (siRNAs) specific for human IFI44 (Thermo Fisher Scientific, s20721 and s225440) or two different siRNAs specific for human FKBP5 (Thermo Fisher Scientific, s5215 and s5216) or with nontargeting (NT) negative control no. 1 (Thermo Fisher Scientific, AM4635). Mouse L929 cells were transfected with an siRNA specific for mouse IFI44 (Thermo Fisher Scientific, s97494) or with NT negative control no. 1. All siRNAs were transfected at a final concentration of 20 nM, using Lipofectamine RNAiMax (Thermo Fisher Scientific), according to the manufacturer’s recommendations.

### RT-qPCR.

mRNA levels of IFI44, FKBP5, IFN-β, IFN-λ1, IFN-induced protein with tetratricopeptide repeats 2 (IFIT2), and IFN-induced protein 27 (IFI27) in human A549, 293T, and HAP-1 cells and in mouse L929 cells were analyzed using total extracted RNA (RNeasy minikit; Qiagen). RT reactions were performed at 37°C for 2 h using a High Capacity cDNA transcription kit (Thermo Fisher Scientific) and random primers and 100 ng of total RNA. qPCRs were performed using TaqMan gene expression assays (Applied Biosystems) specific for human IFI44 (Hs00197427_m1), mouse IFI44 (Mm00505670_m1), human FKBP5 (Hs01561006_m1), human IFN-β (Hs01077958_s1), human IFIT2 (Hs00533665_m1), mouse IFIT2 (Mm00492606_m1), human IFI27 (Hs01086373_g1), and human IFN-λ1 (Hs00601677_g1) genes. Quantification was achieved using the threshold cycle (2^−ΔΔ^*^CT^*) method (58).

### Mass spectrometry.

Human 293T cells were transfected with HA-tagged IFI44 or IFI6 and IFI27 (as controls) expression plasmids (pCAGGS-IFI44-HA or pCAGGS-IFI6-HA and pCAGGS-IFI27-HA, respectively), using DNA-IN (MTI GlobalStem), for 48 h. Cells were lysed in coimmunoprecipitation (Co-IP) buffer (NaCl 100 mM; EDTA 0.5 mM; 20 mM Tris-HCl [pH 7.5], 1 M; Triton X-100 1%; glycerol 5%) containing protease inhibitors (Thermo Fisher Scientific). Then, Laemmli sample buffer (Bio-Rad) and β-mercaptoethanol were added and samples were heated at 95°C for 5 min before SDS-PAGE was performed. Proteins were stained with Coomassie brilliant blue R-250 dye (Thermo Fisher Scientific) and excised from the gel. Gel bands were destained with 50 mM ammonium bicarbonate–50% acetonitrile. After reduction and alkylation, trypsin (Pierce) was added at 10 ng/μl and samples were incubated overnight at 37°C and then extracted using 50% acetonitrile and 0.1% trifluoroacetic acid (TFA). Peptides were desalted, dried in a SpeedVac, and reconstituted in 0.1% TFA–water. Peptides were injected onto a C_18_ column with 1.8-μm-diameter beads (Sepax), using an Easy nLC-1000 high-pressure liquid chromatography (HPLC) system (Thermo Fisher Scientific), which was connected to a Q Exactive Plus mass spectrometer (Thermo Fisher Scientific). Raw data were analyzed using the Mascot search engine (Matrix Science) within the Proteome Discoverer software platform (Thermo Fisher Scientific) and the Swiss-Prot human database.

### Western blots.

Cells were lysed in passive lysis buffer (Promega) and clarified by centrifugation. Cell lysates were mixed with Laemmli sample buffer containing 2.5% β-mercaptoethanol and heated at 90°C for 5 min before SDS-PAGE was performed. Proteins were transferred to nitrocellulose membranes (Bio-Rad) and detected using primary rabbit polyclonal antibodies (pAbs) specific for IFI44 (Abcam ab172499), FKBP5 (Cell Signaling 8245), HA tag (Sigma-Aldrich H6908), FLAG tag (Sigma-Aldrich F7425), phospho-IKKα (Ser176)/IKKβ (Ser177) (Cell Signaling 2078), phospho-IKKε (p IKKε Thr501) (Rockland 600-401-267), and IRF-3 (Abcam ab25950) and mouse monoclonal antibodies (MAbs) against the His epitope tag (Thermo Fisher Scientific MA1-21315), the MYC epitope tag (Thermo Fisher Scientific 13–2500), phospho-IRF-3 (pIRF-3; Abcam ab76493), phospho-IkBα (pIkBα; Thermo Fisher Scientific MA5-15224), IkBα (Abcam ab32518), and actin (Sigma-Aldrich, A1978) followed by incubation with a 1:1,000 dilution of goat anti-rabbit (pAb) or anti-mouse (MAb) IgG antibodies conjugated to horseradish peroxidase (Sigma-Aldrich). Membranes were revealed by chemiluminescence using SuperSignal West Femto maximum sensitivity substrate (Thermo Fisher Scientific), according to the manufacturer’s recommendations.

### Co-IP.

Human 293T cells were transiently transfected with plasmids expressing FKBP5 and IFI44 (pCAGGS-FKBP5-FLAG and pCAGGS-IFI44-HA, respectively) using DNA-IN for 30 h. Alternatively, human 293T cells were transiently transfected with different combinations of pCAGGS-FKBP5-FLAG, pCAGGS-IFI44-HA, pCAGGS-MYC-IKKε, and pCAGGS-MYC-IKKβ plasmids using DNA-IN for 30 h. The total amount of transfected DNA plasmid was maintained at a constant level with empty pCAGGS plasmid. Cells were lysed in Co-IP buffer containing protease inhibitors. Cleared cell lysates were incubated overnight at 4°C with 30 μl of anti-FLAG (Sigma-Aldrich, A2220) or anti-HA (Pierce 26181) affinity resins. After three washes in Tris-buffered saline (TBS) buffer containing 0.1% Tween 20 (for anti-HA resin) or TBS containing 0.1% SDS (for anti-FLAG resin), precipitated proteins were dissociated using disruption buffer at high temperature (95°C) and analyzed by Western blotting as described above using anti-HA (IFI44)-, anti-MYC (IKKε and IKKβ)-, and anti-FLAG (FKBP5)-specific Abs.

### Confocal microscopy.

Confluent human A549 cells on sterile glass coverslips were transiently transfected with pCAGGS plasmids expressing FKBP5-FLAG and IFI44-HA using DNA-IN. At 24 hpt, cells were fixed and permeabilized with 4% paraformaldehyde and 0.1% Triton X-100 for 20 min at room temperature. FKBP5-FLAG and IFI44-HA were detected with murine anti-FLAG and rabbit anti-HA pAbs, respectively. Coverslips were washed 4 times with phosphate-buffered saline (PBS) and incubated with secondary anti-mouse and anti-rabbit Abs conjugated to Alexa Fluor 488 and 546 (Invitrogen), respectively, at room temperature for 45 min. Nuclei were stained using 4′,6-diamidino-2-phenylindole (DAPI; Thermo Fisher Scientific). Coverslips were mounted in ProLong Gold antifade reagent (Invitrogen) and analyzed on a Leica SP8 confocal microscope. Images were acquired with the same instrument settings and analyzed with the Leica software.

### IFN production and signaling assays.

To evaluate the effect of IFI44 on the induction of IFN, human A549 and HAP-1 cells and mouse L929 cells were transfected with siRNAs specific for IFI44 or for the NT siRNA control for 36 h. Then, A549 cells were infected with PR8 (MOI of 3), r3LCMV-GFP/Gluc (MOI of 3), rVSV-GFP (MOI of 0.1), or SeV (MOI of 3) for 24, 42, 24, or 24 h, respectively. Alternatively, A549, HAP-1, and L929 cells were transfected for 16 h with 250, 2,000, and 100 ng/ml of polyinosinic-poly(C) [poly(I·C); Sigma], respectively, using DNA-IN. To assess the effect of IFI44 on IFN signaling, human A549 and HAP-1 cells were treated with universal IFN-α (Axxora) (250 U/ml and 2,000 U/ml, respectively). Total RNA was extracted and RT-qPCRs were performed as described above. Alternatively, HAP-1 WT and IFI44 KO cells were seeded and treated with IFN-α (Axxora) (2,000 U/ml) or transfected with 2,000 ng/ml of poly(I·C) using DNA-IN. At 16 h after treatment, cells were infected with rVSV-GFP (53) for 24 h. Viral titers in tissue culture supernatants were determined in Vero cells as previously described (25).

### IKKε- and IKKβ-mediated activation of innate immune responses.

To measure the induction of IFN mediated by overexpression of IKKε and the activation of NF-κB mediated by IKKβ overexpression, human 293T cells were silenced for IFI44 for 36 h as described above. Then, cells were cotransfected for 24 h with plasmids expressing Firefly luciferase (Fluc) under the control of the IFN-β promoter (pIFNβ-Fluc) (52) (for IKKε overexpression) or pNF-κB-Fluc (Addgene) (for IKKβ overexpression) and a plasmid expressing Renilla luciferase (Rluc) under the control of a constitutive promoter (pRL-SV40; Promega) and pCAGGS-His-IKKε or pCAGGS-MYC-IKKβ plasmid. Cells were lysed for 30 min on ice using a mixture containing 20 mM Tris-HCl (pH 7.4), 5 mM EDTA, 100 mM NaCl, 1% NP-40, and complete protease inhibitor cocktail. Cell lysates were clarified by centrifugation at 14,000 rpm for 10 min at 4°C, and an equal volume of luciferase reporter buffer (Promega) was added. Fluc and Rluc protein expression levels were quantified using a Lumicount luminometer. Levels of Fluc expression were normalized to the levels of Rluc expression. In addition, tissue culture supernatants from pCAGGS-His-IKKε-transfected 293T cells were collected and used to treat A549 cells for 24 h. Then, cells were infected (MOI of 0.1) with rVSV-GFP and GFP intensity was measured using a fluorescence microplate reader (DTX880; Beckman Coulter) and images from GFP-expressing cells were obtained in a fluorescence microscope at 24 hpi.

### IKKβ and IKKε kinase assays.

Human 293T cells were silenced for IFI44 or FKBP5 as described above and, 24 h later, were transfected with pCAGGS-MYC-IKKβ or pCAGGS-His-IKKε, pCAGGS-FKBP5-FLAG, and pCAGGS-IFI44-HA plasmids. The total amounts of transfected siRNA and plasmid DNA were maintained at a constant level with NT control siRNA and pCAGGS empty plasmid, respectively. At 36 hpt, cells were lysed in Co-IP buffer containing protease and phosphatase inhibitors (1 mM Na_3_VO_4_). Cleared cell lysates were incubated for 4 h at 4°C with 30 μl of an anti-MYC affinity resin (Sigma-Aldrich) or with 30 μl of nickel-Sepharose (Ni-Sepharose) Fast Flow resin (GE Healthcare). After three washes in TBS buffer containing 0.1% Tween 20 and two washes in kinase buffer (Cell Signaling), resins were incubated for 1 h at 30°C with 0.5 μg of recombinant IkBα (Sino Biological) (for analysis of IKKβ activity) or IRF-3 (BioPharma) (for analysis of IKKε activity). Then, proteins were dissociated from the resins using disruption buffer and high temperature (95°C) and were analyzed by Western blotting as described above.
