# Supplementary figures and images for: Interferon-Induced Protein 44 Interacts with Cellular FK506-Binding Protein 5, Negatively Regulates Host Antiviral Responses, and Supports Virus Replication
Source: mBio. 2019 Aug 27;10(4):e01839-19. doi: 10.1128/mBio.01839-19 (PMC6712396; doi:10.1128/mBio.01839-19)

# FIGURE S1

**A**

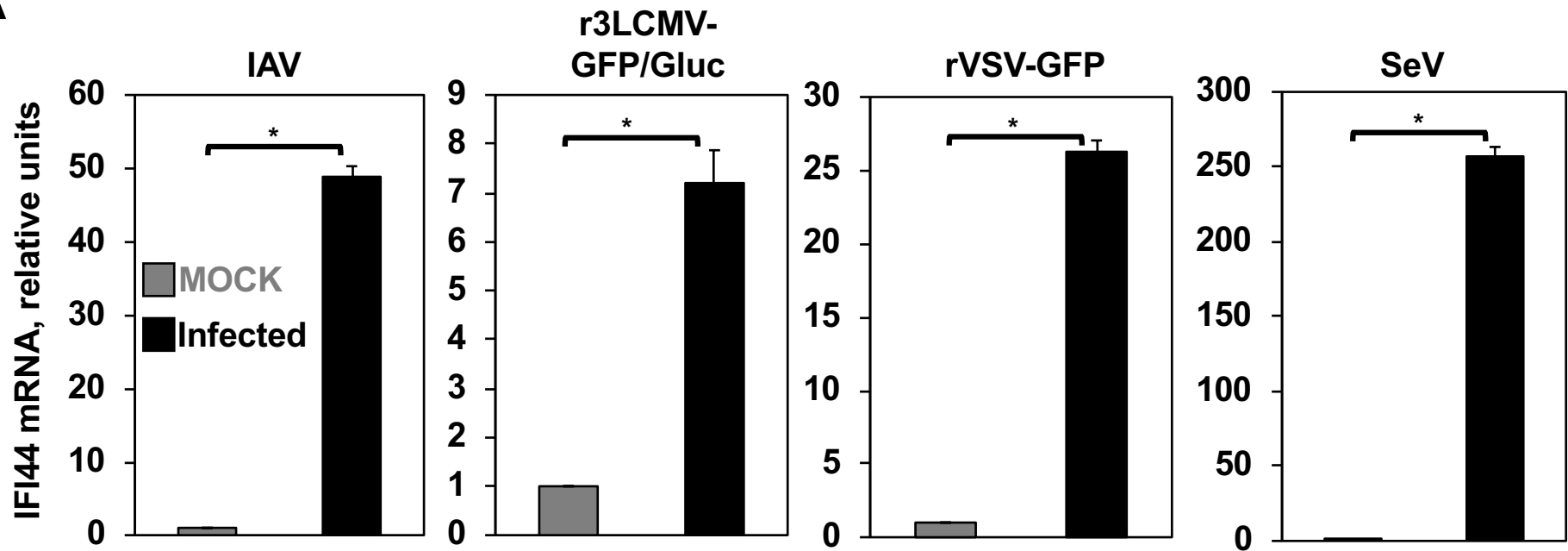

**B**

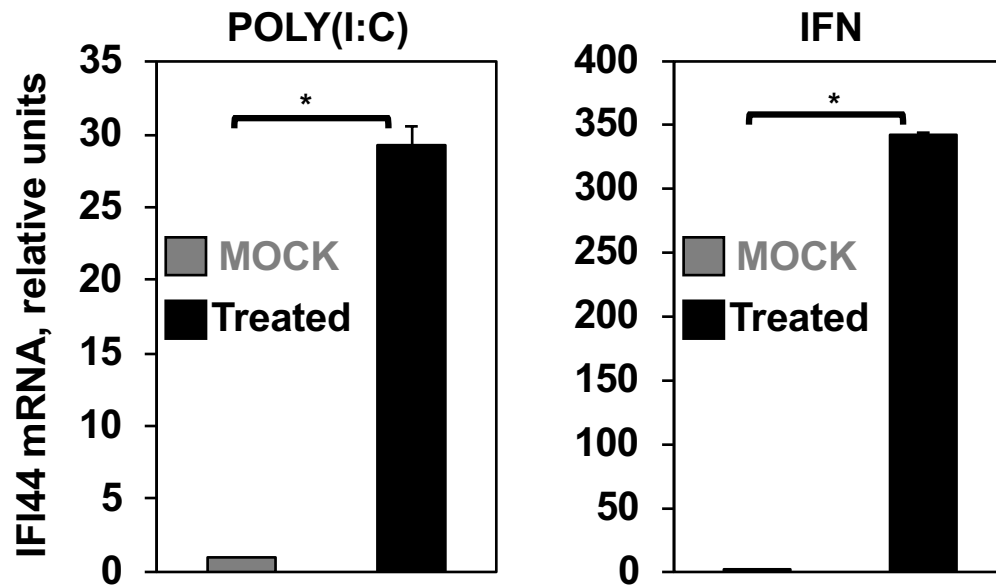

Supplement: FIG S1 [file mBio.01839-19-sf001.pdf]

# FIGURE S2

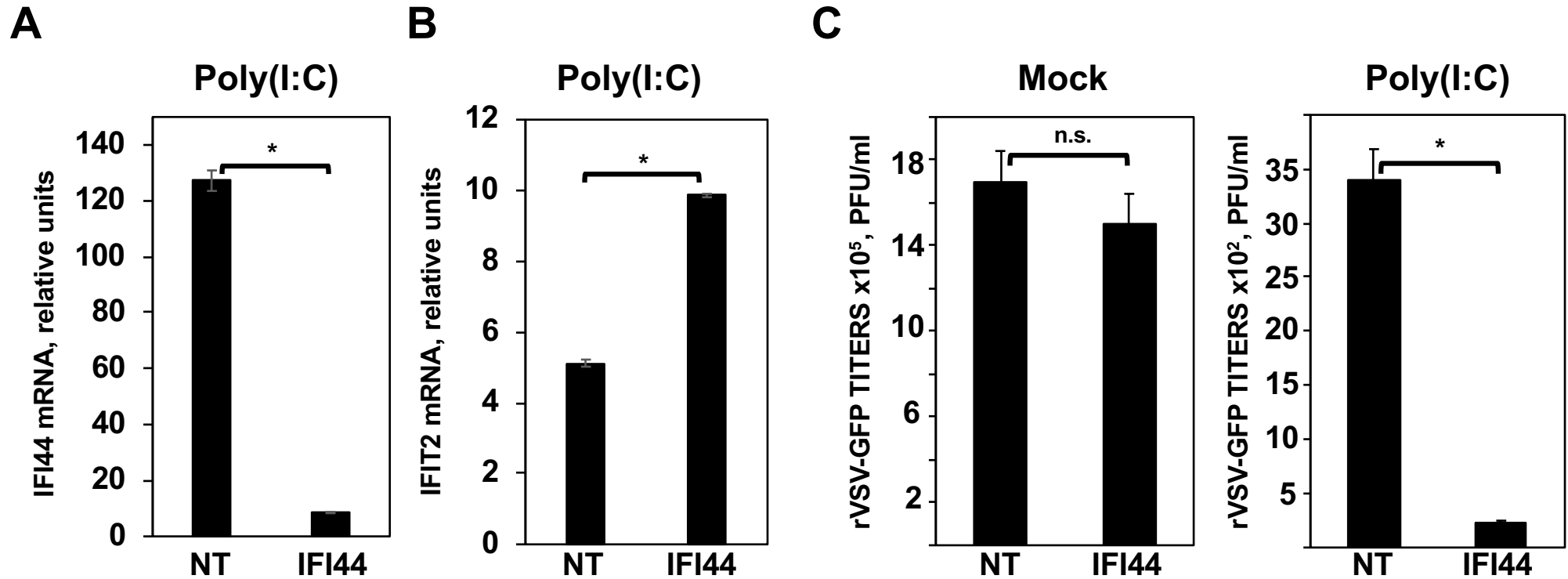

Supplement: FIG S2 [file mBio.01839-19-sf002.pdf]

# FIGURE S3

**A**

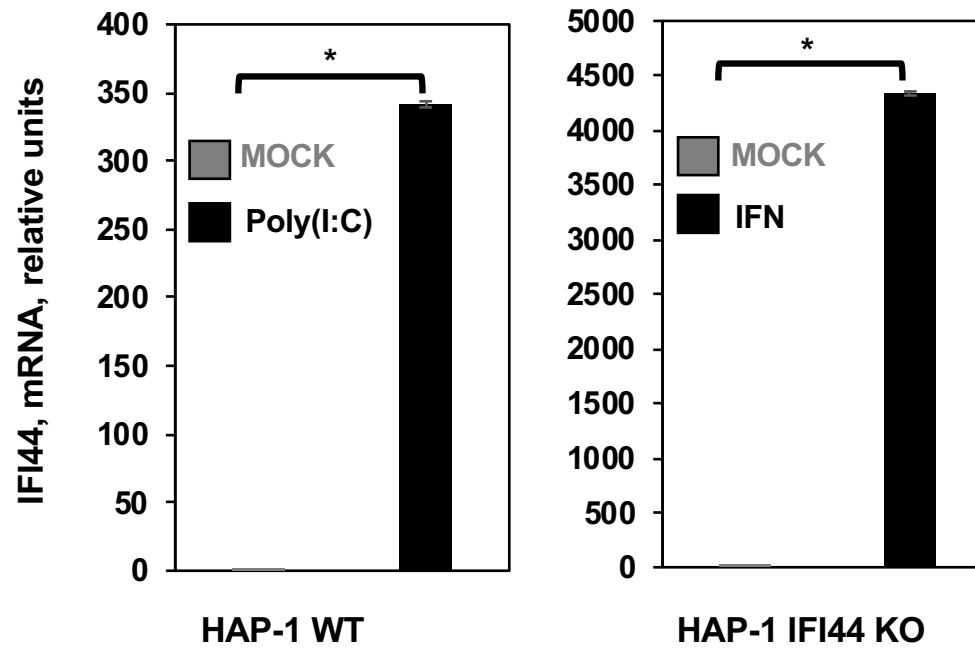

# B

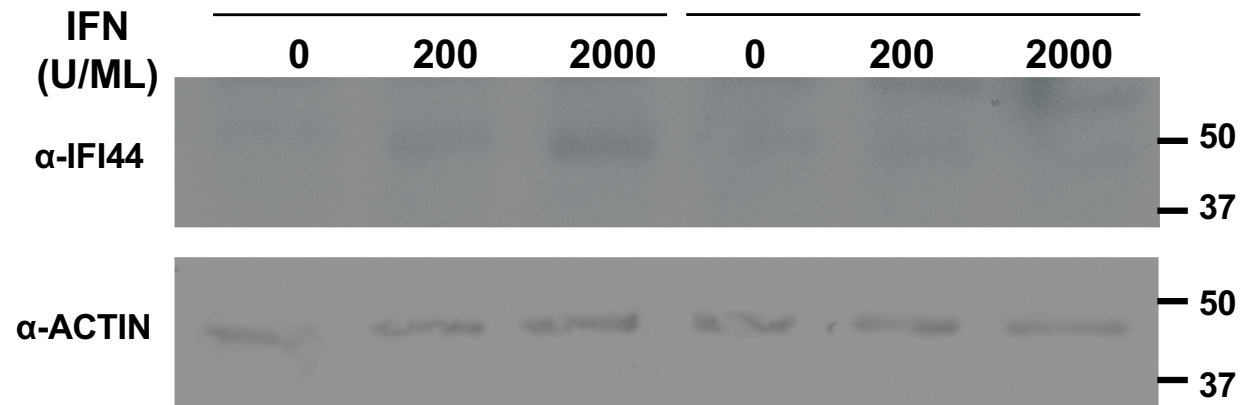

Supplement: FIG S3 [file mBio.01839-19-sf003.pdf]

# FIGURE S4

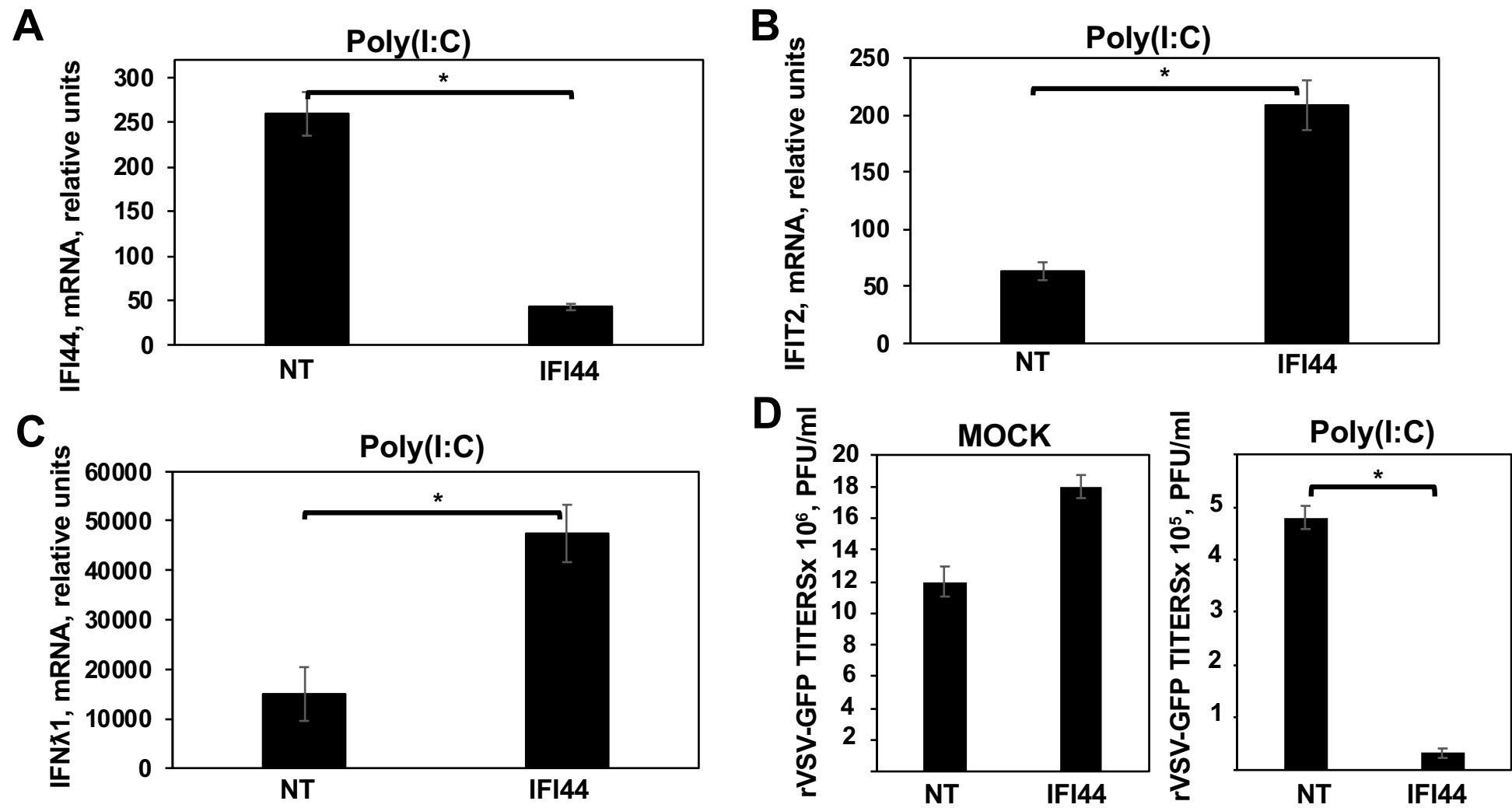

Supplement: FIG S4 [file mBio.01839-19-sf004.pdf]

# FIGURE S5

**A**

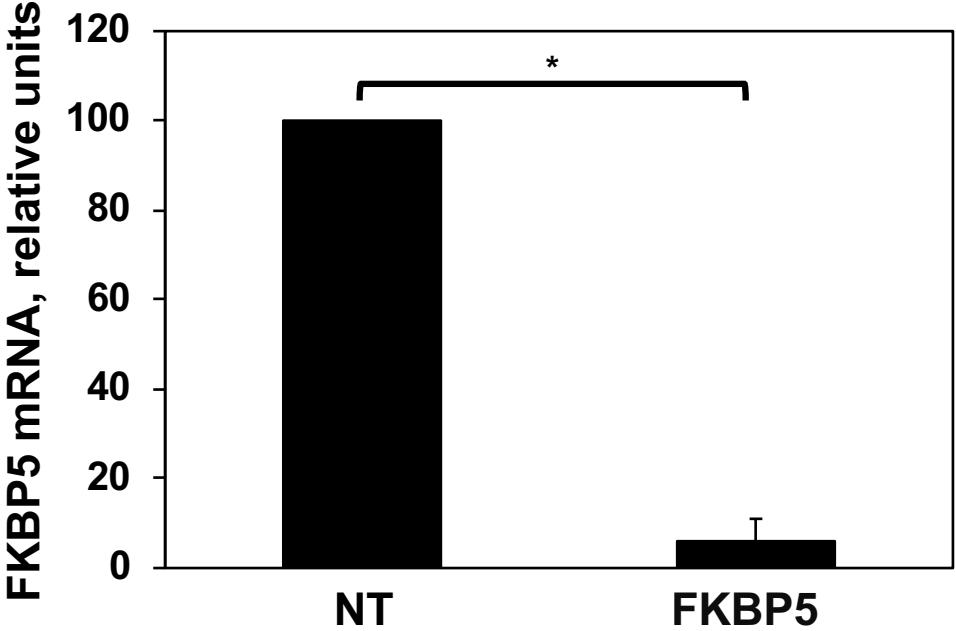

**B**

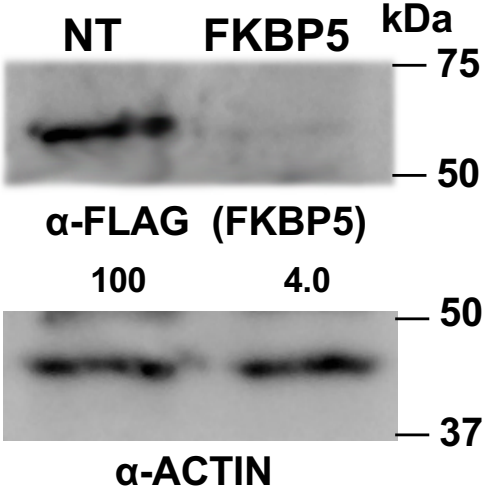

Supplement: FIG S5 [file mBio.01839-19-sf005.pdf]
